# Implementation of Medication Event Reminder Monitors among patients diagnosed with drug susceptible tuberculosis in rural Viet Nam: A qualitative study

**DOI:** 10.1371/journal.pone.0219891

**Published:** 2019-07-22

**Authors:** Dorothy Drabarek, Nguyen T. Anh, Nguyen V. Nhung, Nguyen B. Hoa, Greg J. Fox, Sarah Bernays

**Affiliations:** 1 Faculty of Medicine and Health, University of Sydney, Sydney, Australia; 2 Woolcock Institute of Medical Research, University of Sydney, Sydney, Australia; 3 National Lung Hospital, Hanoi, Viet Nam; 4 Faculty of Public Health and Policy, London School of Hygiene and Tropical Medicine, London, United Kingdom; McGill University Faculty of Medicine, CANADA

## Abstract

**Background:**

Despite the criticality of adherence to tuberculosis treatment, there is paucity of rigorous experimental research exploring the efficacy of interventions to promote adherence and a greater lack of inquiry addressing the integral role of adherence behaviour. The aim of this formative study was to examine the way in which the Wisepill evriMED Medication Event Reminder Monitor (MERM) was used among outpatients with drug susceptible pulmonary tuberculosis.

**Methods:**

In depth interviews were conducted with 20 outpatients receiving treatment from two public healthcare facilities in Thanh Hoa, a rural province in northern Viet Nam. Patients had been enrolled in a randomized controlled trial evaluating the effect of using the MERM device upon adherence for between 1–3 months. The control group used the device without an alert, while the intervention group used the device with a daily alert and scheduled dosing history review.

**Findings:**

All 20 patients interviewed were supportive of using the MERM device. Those able to be at home at the time that their treatment was due (50%) used the device as intended. Patients who worked all reported separating the time when the box was opened from the time at which they ingested their medication. Patients expressed fidelity to the prescribed medication taking time and concerns regarding the portability of the device. Limitations of the study surround the inclusion of a small sample population that did not experience factors that further compromise adherence.

**Conclusions:**

Data recorded by the box did not always accurately reflect usage patterns. The alert in the intervention arm was able to support adherence only in patients who did not work while completing their treatment. MERM implementation can be improved by better aligning prescriber instructions with patients’ daily routines, and increasing the use of adherence data to guide adherence support practices. Healthcare staff need to be aware of potential barriers to optimal use of MERM devices. A rigorous qualitative approach to formative assessment is essential to inform the scale up of new digital technologies.

## Introduction

Viet Nam ranks fourteenth among 20 highest tuberculosis (TB) burden countries, with an estimated TB incidence of 133 per 100 000 population [1, 2]. The infection remains a significant health concern due to both inadequate case-finding [3] and lack of effective strategies with which to increase rates of patient adherence to curative treatment [4]. Incomplete adherence reduces efficacy of treatment, contributes to the development of drug resistant strains of *Mycobacterium tuberculosis*, and is an important factor underlying relapse [5, 6]. Additionally, adherence itself is a complex structural and behavioural issue [4, 7, 8].

Political commitment in Viet Nam has ensured that the National Tuberculosis Program is able to provide TB screening and treatment according to international recommendations. However, limited human resources available at public TB clinics precludes direct daily supervision of treatment by healthcare workers. As in many other high-prevalence settings, TB treatment in Viet Nam is self-administered at home for the vast majority of patients during the maintenance phase of treatment. New affordable technologies promise to enhance patient care through remote adherence support and monitoring. A sustained investigative focus on the use of electronic adherence devices among HIV patients has led to advancements in treatment approaches for antiretroviral therapy [9, 10], however evidence to support the efficacy of such devices among TB patients is limited [11–13].

The WHO Guidelines for Treatment of Drug-Susceptible Tuberculosis and Patient Care (2017 update) recommends the use of digital health interventions as a part of integrated patient care. Medication Event Reminder Monitor (MERM) devices are a new digital technology that records treatment adherence, stores medication and provides alerts at the time treatment is due. These devices aim to enable health facilities to deliver treatment for TB in the community, while closely monitoring adherence [9, 14, 15]. Electronic treatment monitors such as the MERM device offer an indirect method of measurement of adherence to treatment. While having contributed significantly to knowledge of medication taking behavior in various clinical settings, electronic treatment monitor technologies are not able to record whether the patient ingested the medication or what dosage was taken [7, 16]. The claim that the MERM device is accurate in measuring adherence needs to be examined.

A large cluster-randomised trial in China showed that a digital medication monitor box with dosing reminder alert improved adherence to TB treatment [17]. Other studies have shown that patients and healthcare staff find MERM devices acceptable and easy to use [11, 15, 18]. However, behavioural enquiry into how participants use the MERM device is lacking. Such evaluation is a crucial formative step in ensuring optimal use of MERM devices and verifying their accuracy in monitoring adherence to treatment. We conducted a qualitative study as part of a pilot randomised controlled trial (VAS, trial number ACTRN12618000956202) that evaluated the efficacy of the device in improving adherence. Our study aimed to explore patients’ experiences of integrating device use into daily life, in order to better understand the technology and contextual factors which may prevent the MERM system from acting effectively as a support and monitor of adherence.

## Methods

### Study setting

This qualitative study was conducted as a part of the pilot Vietnam Adherence Support (VAS) Trial. The VAS study was a parallel arm randomised controlled trial (RCT) being conducted in Thanh Hoa province in northern Viet Nam. Participants in the main study who were allocated to the intervention group, received a MERM (Wisepill evriMED 1000, Wisepill Technologies, South Africa) that was equipped with a daily medication reminder alert and digital monitor that recorded when the box was opened as a proxy measure for ingestion of medication. Health workers were instructed to use adherence data from individual patients in the intervention group to promote a discussion addressing adherence during the patients’ scheduled monthly clinic visits. Health workers were trained to explore in their monthly discussions with intervention participants, factors contributing to suboptimal-adherence, solutions to improve adherence and adverse event management, if required. If poor use of the MERM device was detected (the MERM was not opened for 20% of days or more in the previous month) then patients were to receive a weekly phone call from VAS study staff and additional support counseling by a health worker. Health workers were also trained in using the MERM device, to check if it was working properly and how to fix it if it was not. Training was provided to health workers twice; once prior to recruitment into the main study and again after 50 participants were recruited. In the control group, patients received a MERM which recorded when the box was opened, but did not alert the patients to the timing of their doses. Health workers were not provided with data about control patient adherence for this group and although monthly check ups were scheduled, they did not include discussions based on the digital adherence report. According to the report, a day without a record of box opening indicates one missed dosing event [19]. Due to the MERM device’s inability to measure ingestion of medication, intended use of the device involved opening it at the scheduled medication taking time and ingesting medication at the same time.

Participant interviews for the qualitative study were conducted either in an unoccupied formal meeting room in a Yen Dinh District TB Clinic, or an unoccupied formal meeting room at Thanh Hoa Provincial Lung Hospital. Only the interviewers and the translator were present during interviews which were scheduled to coincide with monthly check-up and medication replenishment. Participants who requested interviews outside of the health facilities met with qualitative study staff in other locations in the community.

### Study population

During December 2017 and January 2018, VAS study staff invited consecutive patients enrolled in the RCT to participate in in-depth interviews to evaluate the acceptability and feasibility of the MERM device intervention. Participants were invited during their monthly check up visit. Eligibility was subject to being 15 years of age or above with newly diagnosed microbiologically confirmed pulmonary tuberculosis, taking only standard oral therapy for tuberculosis as an outpatient, with at least four months of treatment remaining. Exclusion criteria included subjects diagnosed with multi-drug resistant TB prior to enrolment and those with severe mental illness. Enrolment into the VAS RCT was voluntary for patients receiving treatment at Thanh Hoa Lung Hospital and Yen Dinh TB Clinic. These eligibility criteria for the qualitative study were the same as for the VAS RCT.

### Data collection and analysis

Interviewers asked opened questions drawn from a topic guide of key ideas flexible enough to adapt the form and sequence of enquiry to the experiences of each participant. The interviews were conducted jointly by two female researchers, one Vietnamese, one Australian (DD), who were not known to any of the participants prior to the study. The chief investigator provided training and supervision to the research team throughout. The researchers did not hold any personal interests that could bias the study results. In line with appropriate ethical considerations, given the sensitivity of reporting non-adherence and to minimise social desirability bias, an emphasis was placed on the independence of the qualitative study team from clinic staff and the confidentiality of all interview accounts. Interviews lasted between 45 and 75 minutes. The interviews were audio-recorded and conducted in Vietnamese with simultaneous translation provided to the Australian researcher who posed additional questions as appropriate, with due care being paid not to disrupt the interview dynamic. Field notes were also taken. Recorded data were transcribed verbatim by an external transcription service. Translation into English was done by two Vietnamese researchers who checked each other’s translations for accuracy. Personal identifying details were removed from the data at transcription stage.

A discussion was held immediately after each interview between researchers and translator to clarify concepts raised. Daily discussions were held during the data collection process between the interviewers, translator and chief investigator to consider emerging analytical ideas, opportunities to refine the interview guide and approach, and to identify theoretical saturation. Thereafter, preliminary codes were developed inductively from interview summaries and agreed upon by the Australian researcher and chief investigator. A coding framework was developed and applied to the data. Then, through ongoing coding, case comparison, memo writing and analytical discussions [20] involving all members of the qualitative research team, key themes and interpretations were identified. Data were managed using Microsoft Excel. Feedback from the qualitative study was given to VAS study staff who as a result implemented a more thorough procedure for monitoring monthly adherence discussions. As the RCT was ongoing, qualitative study participants did not receive feedback on findings.

### Ethical issues

Ethical approval (no. 2017/226) for the RCT and qualitative sub-study was granted by the HREC of the University of Sydney and the National Lung Hospital, Vietnam. Parental or guardian consent was not sought for one 15 year old participant, this was approved by the HREC and the National Lung Hospital ethics committee. Consent was discussed with an illiterate participant and their marking of the consent form was witnessed by the researchers and translator. All participants of the qualitative sub-study provided signed informed consent and received a modest financial reimbursement in line with local research practice.

## Results

Of 24 consecutively patients enrolled in the RCT, 20 (83.3%) agreed to take part in the qualitative study. Each participant was interviewed once. Reasons for non-participation included patients working in a province different from the clinic (travel distance) or being uncontactable.

We enrolled 17 males and 3 female patients. This distribution reflects both the profile of participants in the RCT, and the male predominance among patients with TB in Viet Nam [21]. At the time of interview, participants had been using the MERM device for between five to twelve weeks. Patients recruited to the study were of different socio-economic backgrounds and resided in rural and peri-urban areas in Thanh Hoa Province. Some participants experienced risk factors for non-compliance such as illiteracy, poverty and distance to TB clinics [22], factors that the MERM intervention aims to overcome. One elderly lady was illiterate; no participants described experiencing abject poverty, however there were three participants who reported experiencing ongoing financial hardship, which affected their capacity to rest, as they could not afford not to work throughout their treatment, and to eat well. Another participant reported receiving government health insurance which is received only by the very poor; three participants had to travel over 20 km to reach the hospital or clinic, with the furthest distance travelled being 60 km. There were no participants who experienced more than one risk factor for non-compliance. Prior to commencing treatment within the public tuberculosis control program, participants had sought care from private and public commune level clinics, district hospitals and pharmacies and public tertiary hospitals. Table 1 shows the characteristics of study participants, including their occupations.

**Table 1:** Qualitative sample overview n = 20.

Age range	No.	MERM with alert (I)	MERM without alert (C)	Female	Male	Working away from the home	Working within the home or vicinity of the home or unemployed
15–19	1		1		1		student
25–29	3		3		3	fabric factory supervisor, financial manager	college graduate
30–34	1	1			1	farmer	
35–39	1		1		1	truck driver	
40–44	1	1			1	stone mason	
45–49	5	4	1	2	3	farmer/builder	farmer, fisherman, fish trader, printing factory worker
50–54	1	1				building site inspector	
55–59	4		4		4	builder	farmer/builder, farmer, farmer
60–64	2	1	1		2	farmer, commune leader (retired electrician)	
70–74	1		1	1			rice farmer
Total *n (%)*	20 (100)	8 (40)	12 (60)	3 (15)	17 (85)	10 (50)	10 (50)

Note: No participants in the 20–24 years and 65–69 years age groups were enrolled.

I, intervention; C, control

The MERM device was reliable, in that it did not falter in its ability to send digital signals over long distances and in only two instances did the audio alert sound at unexpected times. For one participant in the control group, upon commencing to use the device the alarm erroneously sounded at midnight and for one participant in the intervention group the alarm had been set by study staff one hour earlier than the medicine taking time prescribed by the doctor. All participants considered the box to be useful, citing various reasons including keeping the medication dry, safe and neat and acting as a reminder. Among those who said it was useful in supporting adherence, there was variation in whom they considered it to be useful for: either for themselves and others or older people only and not themselves. Several participants noted that the box might be more beneficial as an adherence reminder tool for people of older age. However, we found that location of work, away from the home or within the home environment, rather than age, were the main determinants of how the MERM box was used and its related efficacy. The association between location of work and use of the MERM device emerged from the data as the strongest thematic pattern in our analysis. Participants who did not use the box as intended all worked outside of the home on a daily basis, including those who were poor and needed to work throughout their treatment. Those who could stay home during their treatment and use the box as intended were those who were self-employed as farmers working on land adjacent to their homes or those who were unemployed during treatment, the latter included those who no longer worked due to older age. Those who were unemployed during their treatment expressed that the box would not be as useful if they needed to leave the home for work each day.

### Participant use of the MERM device

The in-depth interviews revealed that ways in which participants used the MERMs were instrumental in shaping the intervention’s efficaciousness as an accurate monitor and support of adherence. Upon recruitment to the study, all participants received training from VAS study staff. Participants reported that they had been instructed to open the MERM box and take their medication at 8, 8.30 or 9am every day. Some participants described an additional instruction of either 30 minutes before food or one hour after food, this was an instruction given by treating doctors and not a requirement of the study. For those in the intervention arm, the alert on the MERM box was set to coincide with the time that the doctor had prescribed for medication taking or, as for two participants, the time at which the patient thought it would be most convenient to open the box (but not to take medications). These patients customised the time at which their alarm sounded by asking VAS study staff to change it. It was not clear however, if all participants were aware of such a possibility. Opening the MERM box and ingesting medication at the same time relied on the participant being in the vicinity of the device at medication taking time. In recounting how, participants used the MERM device, it became clear that there were two distinct patterns of use.

Those who used the box as it was intended all or most of the time tended to be those who were able to stay at home for the majority of each day. This was because when it came to the time to take their treatment they were close to the box, enabling them to open it, extract their pills and immediately ingest them. The digital recording of the box being opened was therefore an accurate record of treatment consumption and thus adherence. However, among all 10 participants who needed to leave their house every day for work, the opening of the box did not indicate immediate consumption of the pills. Instead, participants would open the box early in the day before leaving home and carry their daily dose in a more transportable and practical way, such as in their pocket or bag. No participant who left their house daily used the box or took their medication as the MERM device manufacturers intended. Participants who were away from home for more than one day would take as many doses out of the box as days they were away.

A: “Everyday, in the morning ‥ around 7 am, I open the box and bring the drug along. Except some days that I have to go for two days, I cannot open the box on the next day …

Q: Yes, so for those two day trips, how do you do with your drug?

A: I leave the box at home, I just take medicine, put it in the nylon bag and bring it along.” **(P06-TH-C- working)**

**P16-C** took his medication to work with him and there he relied on his own entirely separate reminder system in the form of a mobile phone alarm. Younger study participants tended to set their own alerts on personal electronic devices, demonstrating utility of multiple digital technologies as adherence supports.

A: “… I set the [phone] alarm at three different times, 15 minutes before, 5 minutes before and 5 minutes later. It reminds me continuously to avoid the situation when you’re too busy.” **(P16-TH-C-working)**

For most participants in the intervention arm, opening the box before leaving for work also meant opening the box prior to the alert sounding, rendering the MERM’s reminder alert feature ineffective in supporting adherence. Upon receiving their MERM box, **P12-I and P05-I** had their alert set by study staff to sound at 6.30am and 7am respectively, due to work schedules. However, both participants continued to carry the medication to work and ingest at 9.30am and 9am. No subsequent or other adjustments were reported by intervention arm participants. Only **P02-I** mentioned to a healthcare worker that his routine consisted of separating the time at which he opened the MERM box from when he ingested medication. He reported that the healthcare worker said this method of using the box was allowed.

Q: “You open the box at 6:30?

A: Yes. At around 6:30 and 6:40 when I leave the house.

Q: So‥ why are you not taking them right in the morning but waiting until…

A: Because the doctor said to take them at around 9:00 and 9:30 in this period…” **(P12-TH-I-working)**

We examined the reasons behind why participants who were away from their home during the day developed this behavioural pattern of twinned treatment schedules. We found that it was driven by two key concerns. The first related to the physical attributes of the box: it was too big and inconspicuous for participants to carry with them.

A: “…I opened the box and took medicine (out of the box) in advance… I am afraid if I brought it along. Every time when I go out, I often take this bag and then put it in a travel bag with a ziplock.” **(P03-YD-I-not working)**

Nineteen of 20 study participants did not did not take the box out of their home, the exception being **P14-C**, albeit describing doing so as inconvenient.

A: “… for a young guy, the box is also inconvenient because it is a little big for us to bring it along when hanging out or travelling … it is cumbersome to take along.” **(P14-TH-C-working)**

The MERM box’s lack of practical portability is important to note, in particular that its portability is a feature identified by the manufacturer as key to its usefulness [23]. It is also pertinent that some patients described the box as not big enough, in that it did not fit both their TB medications and the prescribed supplements and vitamins they took daily to counter the potential toxicities of the medication.

The second driver of how the box was used among study participants who worked, reflects a nexus of values which shaped the way that participants perceived and interacted with their prescribing clinicians. Participants described having an unquestioning trust in their doctor and in the treatment’s ability to cure them of their illness.

A: “In my opinion… if you have an illness and you don’t follow the treatment plan of doctors then you shouldn’t go to the hospital at all… Now you’re a doctor. You tell me to take this, take that, I trust you and if then I don’t do things like you said… it will become useless, then what’s the point of going… we should obey.” **(P05-TH-I-working)**

A: “Yes, generally if I follow the treatment plan strictly, there wouldn’t be any problems… Lung TB can totally be cured.” **(P16-TH-C-working)**

We found that congruent with participants’ unquestioning trust in their doctor, they adhered precisely to the doctors’ instructions for when medication should be taken. These instructions had not been tailored to fit within the individual’s existing routine. However, participants went to considerable efforts to comply with the recommended time for taking medications. As a result, the act of ingesting the pills became independent of the MERM box in both time and location. In some cases, participants had inadvertently and unnecessarily complicated their treatment routines by attempting to accommodate both their doctor’s instructions and their existing habits and routines.

### Impact of patterns of use on efficacy

**P16-C** is an illustrative case which highlights the limitations in the way that the MERM box was used and the related challenges this poses for how to interpret the digital monitoring data. This 29 year old man’s digital adherence report, spanning three months, showed that the box was opened only five or seven times per month. In his interview however, he explained his meticulous daily routine to ensure that he did not forget any doses (as quoted above).

A: “…I was using the box normally but only during the time of my treatment at home. Since I started going to work, I haven’t been using it that much because I cannot bring it along. It’s very cumbersome.

Q:‥ so do you take medications out for one day only or for few days?

A: Around three or four days… I put them in my backpack just in case I forget so I just put them in there.” **(P16-TH-C-working)**

For **P16-C**, the intervention became ineffective as both a support for adherence and adherence monitor. Without additional corroboration the resulting digital record erroneously indicated that he was a poor adherer.

## Discussion

This qualitative study documents use of the MERM device as being viewed positively by outpatients receiving treatment for drug susceptible tuberculosis, and for those able to stay at home during treatment, the device appears from our analysis to be working as intended. However, due to how the MERM device came to be used among study participants in both study arms whose daily routines did not support its proposed pattern of use, the MERM device did not always fulfil its intended role as a support for adherence. Nor did the intervention provide an accurate record of adherence for these participants. Digital data reports frequently underestimated adherence and masked the complications of separating the times at which the box was opened, and medication was taken. Additionally, despite research staff receiving adherence data from the devices, patients in the intervention group did not report that this information had been raised in their discussions with a health worker. This represented a missed opportunity to use data provided by the MERM system, which is designed to deliver information to health practitioners and guide their adherence support. It is possible that health workers were not appropriately aware of the value of the generated adherence report as a tool with which to guide counselling, or that the report would not always accurately reflect patterns of use due to its nature as an indirect measure. Future studies should include interviews with health staff to better understand the interactions between health providers’ understanding of a technology used to support adherence to treatment and their patients’ uses of it.

The MERM intervention provides the greatest benefit if patients are able to ingest medications at home when the adherence alert sounds. This can be achieved by customising alerts to sound at times at which patients wake, sleep or eat. Such an approach would overcome the identified limitations of the device, and would preclude the need for alternative portable methods of storage for patients who leave home early. An alternative strategy for patients unable to consume their medications at home would be the use of smaller, portable, devices. However, their capacity is not sufficient to store the large volume of medications required for a month of treatment. Our study suggests that the benefit of using data from MERM devices will be greatest when doctors explicitly discuss with patients how use of the device can best fit into their daily routines, and incorporate data from the devices into patients’ monthly clinic reviews. Such interaction by health staff could also result in improved adherence through a ‘Hawthorne’-like effect, and engender a greater sense of support [9, 10].

The limitations of this study should be noted. Study subjects were not representative of all patients taking treatment for TB in Viet Nam. We did not identify patients experiencing factors known to substantially increase risk of non-adherence such as substance abuse, mental illness, HIV co-infection or MDR-TB [24, 25]. As such our conclusions have limited generalisability. Information regarding substance abuse was collected in a baseline questionnaire carried out by study staff and completed by all study participants and HIV co-infection status was determined from participant hospital or clinic medical records. Further enquiry of the kind we have carried out is necessary among groups of patients whose circumstances further compromise opportunities for adherence [26]. In addition, given the relatively small sample size, the findings may not be representative of how all drug-susceptible pulmonary TB patients use the MERM device, nor is the inconsistent predictive validity of digital dosing histories an original finding [7, 9, 27]. Similarly, a small sample size that included a small proportion of women limited the ability of our analysis to detect gendered patterns of use. However, the sample elucidated several important drivers of ineffectual use of the MERM device in a consecutively recruited study population, which to our knowledge have not been reported previously.

This study has several important public health implications for the use of a MERM system in resource-limited settings. We have shown that implementation may differ substantially from the recommended approach. Early evaluation of patient and health worker behaviours and beliefs following implementation of this technology in a new setting will be essential in optimising its acceptability and clinical impact. Secondly, we have identified substantial inter-patient variation in the way in which these devices are used. Clinicians ulitising MERM devices will need to be aware of potential barriers to their optimal use and orient their approach towards a patient-centred model of care. This will require training staff to be able to detect ineffectual device use and health worker practices, and to understand the nuances of how digital technologies operate and affect patient behaviours (and vice versa). Clinicians need to initiate proactive discussions with patients to elicit patterns of and approaches to use not detectable by the indirect measure that MERM device provides. Additionally, it is important to demonstrate to clinicians the potential of a technology such as the MERM to assist in targeting support and limited resources when the device is not being used as intended. Finally, the introduction of new technologies alone is just one part of a broader approach to adherence support. Technological innovations must be accompanied by sustainable health system strategies to address and overcome diverse barriers to treatment completion. Conceiving of these devices purely as ‘monitors’ of patient behaviour fails to recognise the important collaborative nature of adherence support, as reflected in recently revised WHO treatment guidelines [12].

Insights gained from our study provide important guidance for the scale-up and integration of MERM devices into care in resource-limited settings such as Viet Nam. Lessons learned from this study may also be applicable to patients taking and health staff administering other long term treatment regimens such as antiretroviral therapy [13] in similar settings. Lastly, our behavioural investigation directs new angles of enquiry into how the MERM device and system can positively influence adherence behavior and further inform the development of patient-centred strategies utilising digital technologies.

## Supporting information

S1 File Topic guides. (DOCX)
