# Supplementary material for: Implementation of Medication Event Reminder Monitors among patients diagnosed with drug susceptible tuberculosis in rural Viet Nam: A qualitative study
Source: PLoS One. 2019 Jul 22;14(7):e0219891. doi: 10.1371/journal.pone.0219891 (PMC6645511; doi:10.1371/journal.pone.0219891)
Supplement: S1 File — (DOCX) [file pone.0219891.s001.docx]

# **VDOT2 Qualitative substudy**

**Indicative topic guides**

**In-depth interview**

| KEY AREAS OF INVESTIGATION | EXAMPLE QUESTIONS |
| --- | --- |
| Reasons for participation in VDOT2 study | Can you tell me how you came to be involved in the VDOT2 study?  Why did you choose to take part? |
| Personal | |
| Attitudes and feelings about your initial diagnosis and proceeding diagnoses if relevant and changes since  Knowledge of TB and sources of information | As much as you can remember, can you tell me about your initial thoughts, feelings or reactions upon learning your diagnosis.  Have they changed since then?  What do you think is the cause of your TB?  What are the possible causes of other peoples’ TB?  How do you know that?/Where did you get that information from? |
| Individual’s sense of relative health | How do you feel physically now? How does this compare with when you first started treatment? |
| Attitudes towards TB treatment and need for compliance | Can you tell me a bit about what you feel about having to take the pills?  What are your thoughts on what might happen if you do not take the pills as prescribed?  What is your motivation to adhere to the treatment regimen? |
| Device | |
| Experience of using the device and any changes over time, including (changing) degree of comfort with the technology | Can you tell me about what pills you need to take during the day and how you take them?  How often do you add medication into the box?  Do you know how to recharge the box?  Can you talk me through whether and how you used it yesterday?  In the past, how did you remember to take the drug daily without the box/box with the alarm? |
| Management of TB treatment and the device alongside other treatment if there are co-morbidities | How do you manage taking the prescribed vitamins to assist with side effects of TB treatment?  Are you trying any other treatments for TB other than those given by free by the Vietnamese TB program?  Are you taking any other medication other than for this condition?  Can you talk me through how you take all your pills during the day? |
| External | |
| Experience of and attitudes towards adherence and missed doses – is there stigma associated with non-adherence to treatment? | If you miss a dose, what do you do? Do you tell anyone? If so, what was their reaction? If not, why? |
| Experience and attitudes towards other people’s awareness of their TB diagnosis and treatment | Who knows about your diagnosis? Can you tell me more about how that came about and their reaction? Why do you think their reaction was as it was?  (Household, extended family, friends, co-workers.) |
| Use of the MERM box | How has your experience been using this box?  If you don’t use the box, why?  Who knows that you are using the box for TB treatment? What was their reaction? Why do you think this was?  Do you tell others about the box? Why/why not? If somebody sees your box and enquires about it, what would you say?  Do you want to introduce the box to others? Why/why not?  Have you every had any problems with the box?  Is there any thing you would change about the box? |
| Role of healthcare provider in patient’s experience of use of the device | What do you think about the information that is given to you about TB and the treatment? What kind of information/support did you receive?  What do you think about the instruction you received on how to use the box?  If you have some issues with the box, who will you ask? What do you expect from HCW when you ask/call them? (Counselling, inform about side effects or ask about box’s issues..) |
| Self-identified adherence support needs, including the use of technology, health clinic facilities and staff, family, and/or close persons, peer support. | What do you think you would need to be able to take your treatment consistently each day?  What are your thoughts on discussing issues with other patients? |
